# miRNA Profiles of Canine Intestinal Carcinomas, Lymphomas and Enteritis Analysed by Digital Droplet PCR from FFPE Material

**DOI:** 10.3390/vetsci10020125

**Published:** 2023-02-06

**Authors:** Alexandra Kehl, Mario Valkai, Anna-Lena Van de Weyer, Maria Brockmann, Katja Steiger, Benjamin Schusser, Heike Aupperle-Lellbach

**Affiliations:** 1 LABOKLIN GmbH & Co. KG, 97688 Bad Kissingen, Germany; 2 Comparative Experimental Pathology, School of Medicine, Technical University of Munich (TUM), 81675 Munich, Germany; 3 Reproductive Biotechnology, School of Life Sciences Weihenstephan, Technical University of Munich (TUM), 85354 Freising, Germany

**Keywords:** lymphoma, carcinoma, enteritis, intestine, dog, miRNA, ddPCR

## Abstract

**Simple Summary:**

Carcinomas and lymphomas are the most common intestinal tumours in dogs. To distinguish them from each other and from intestinal inflammation, non-invasive biomarkers such as microRNAs would be helpful. In this study, miRNA expression of two oncogenic miRNAs (miR-18b, 20b), two tumour-suppressive miRNAs (miR-192, 194) and two potential biomarkers (miR-126, 214) in carcinomas, lymphomas, enteritis and normal tissue of the small and large intestine were measured. Significant shifts in miRNA expression patterns were observed between the groups. However, the expected dysregulation of miR-18b, 20b, 126 and 214 was not found in all tissues. Further studies are needed to clarify the role of different miRNAs in various types of tissue and cancer.

**Abstract:**

Most canine intestinal tumours are B-cell or T-cell lymphomas or carcinomas. They have to be distinguished from cases of enteritis. Non-invasive biomarkers such as miRNAs would be a step towards faster diagnosis. The aim of this study was to investigate shifts in miRNA expression in tissue samples collected from cases of enteritis, carcinoma and lymphoma of the small and large intestine to better understand the potential of miRNA as biomarkers for tumour diagnosis and classification. We selected two oncogenic miRNAs (miR-18b and 20b), two tumour suppressive miRNAs (miR-192 and 194) and two potential biomarkers for neoplasms (miR-126 and 214). They were isolated from FFPE material, quantified by ddPCR, normalised with RNU6B and compared with normal tissue values. Our results confirmed that ddPCR is a suitable method for quantifying miRNA from FFPE material. Expression of miR-18b and miR-192 was higher in carcinomas of the small intestine than in those of the large intestine. Specific miRNA patterns were observed in cases of enteritis, B-cell and T-cell lymphoma and carcinoma. However, oncogenic miR-18b and 20b were not elevated in any group and miR-126 and 214 were down-regulated in T-cell and B-cell lymphoma, as well as in carcinomas and lymphoplasmacytic enteritis of the small intestine.

## 1. Introduction

Intestinal tumours in dogs include lymphoma, carcinoma, gastrointestinal stromal tumour and leiomyo(sarco)ma [1,2,3]. One of the most important clinical methods in the diagnosis of intestinal neoplasms is the ultrasound examination, but differentiation from inflammatory masses can be challenging [4]. Cytological examination may be helpful [5], but often laparotomy and histopathological examination are necessary to confirm the diagnosis. In general, cancer screening tests [6], liquid biopsy techniques [7], including cell-free DNA in plasma [8,9], and microRNA (miRNA) [10] are the focus of current research in veterinary oncology to improve the pre-operative diagnosis.

MicroRNAs are small non-coding RNAs which regulate the expression of genes by translational suppression or enhancement, and are involved in cell development and apoptosis in healthy individuals [11]. There is strong evidence that miRNAs play a role in oncogenesis by having tumour-suppressive or oncogenic effects [12,13]. To further elucidate the functions of miRNA in the development of cancer, the miRNA profiles of healthy and cancer tissue are compared to evaluate the difference in occurrence and expression level of the different miRNAs. This can help to understand the role of miRNAs in oncogenesis, leading to potential targets for therapeutic agents [14].

Tumour cells not only show a different miRNA profile, but also release these miRNAs into the blood stream, stabilised by binding proteins or packed in exosomes and microvesicles. These “circulating miRNAs” probably reflect the existence and the amount of tumour cells [14]. In recent years, miRNAs have gained greater attention as they seem to be able to serve as biomarkers for diagnosing and monitoring tumours [14,15].

In dogs, knowledge regarding the miRNA profiles of healthy intestinal tissue is limited, as well as regarding tumours of the small or large intestine. Intestinal T-cell lymphoma showed down-regulation of miR-192, 194, 141 and 203, while miR-20b, 18b and 363 were up-regulated [16]. Up-regulation of miR-18b seems to be involved in the development of colorectal cancer in humans by promoting cell proliferation and migration [17].

Circulating miR-20b was described as a potential biomarker for distinguishing gastrointestinal cancer from chronic inflammatory enteropathy in dogs, which probably reflects the important role of miR-20b in intestinal diseases [18]. Decrease in miR-192 expression (together with the shift in other miRNAs) was described in the mucosa and serum of dogs with large intestinal inflammatory bowel disease [19]. In humans, a decrease in miR-192 is associated with growth and metastasis of colorectal cancers [20]. Suppression of miR-194 was correlated with the growth of intestinal tumour organoids [21]. In human serum samples, a decreased miR-194 level was described as a suitable biomarker [22] and a predictor for colorectal adenomas [23]. Circulating miR-126 and miR-214 have been described as general biomarkers for neoplastic diseases in dogs, including endothelial and epithelial tumours. Both miRNAs have been described as being over-expressed in individuals with different tumour types [10,24].

In our study, we investigated the expression of the abovementioned miRNAs (miR-192, 194, 18b, 20b, 126 and 214) in different canine intestinal tumours compared with enteritis and healthy tissue. We chose two miRNAs with suspected oncogenic function (miR-18b, 20b) and two with a possible tumour-suppressive function (miR-192, 194). The remaining two miRNAs (miR-126, 214) were tested because they have been described as potential biomarkers for neoplastic diseases in general.

The aims of this study were: (1) to evaluate potential differences in the expression of the selected miRNAs between normal small and large intestinal tissue; (2) to investigate the expression changes of these miRNAs in canine small and large intestinal carcinomas, T-cell lymphomas of the small intestine, B-cell lymphomas of the large intestine and enteritis compared with healthy tissue; and (3) to assess the potential of these miRNAs as discriminators between the defined groups.

## 2. Materials and Methods

### 2.1. Selection of Samples

The canine intestinal samples collected for routine diagnostics were divided into four groups: (1) controls: normal tissue of the small and large intestine; (2) different types of enteritis in the small and large intestine; (3) lymphomas of the small intestine (T-cell) and the large intestine (B-cell); and (4) carcinomas of the small and large intestine (Table 1). All samples were randomly chosen from routine material and needed to fulfil the following inclusion criteria: good sample quality (minimum size 0.5 cm, no artefacts), a definite diagnosis and fitting into the predefined groups. Samples were excluded, if the samples were too small, a definite diagnosis was not possible or if the samples did not fit into any of the groups.

Preparation of formalin-fixed tissue samples, including macroscopic description (e.g., size of the mass), was performed according to trimming guidelines [25]. All samples were embedded in paraffin wax and cut into 2–3-µm-thick slices, which were stained with haematoxylin-eosin (HE). Where necessary, special stainings (e.g., Ziehl-Neelsen, giemsa or PAS-reaction) were performed according to standard protocols [26]. Samples were collected between 2014 and 2022.

Grading and characterisation of enteritis were performed according to the literature [27]. Carcinoma subclassification (acinar, papillary, mucinous, signet-ring, undifferentiated) followed the WHO classification [28]. In cases of mixed growth patterns, the predominant type (at least 75%) was reported. Intestinal T-cell lymphoma and extranodal marginal zone B-cell lymphoma of mucosa-associated lymphoid tissue (MALT lymphoma) were classified as described by the WHO (Head et al. 2003). The size of the neoplastic lymphocyte population was reported as small-cell, intermediate-sized and large-cell lymphoma, as defined by the WHO [29]. The mitotic count was evaluated in 10 high power fields (hpf, FN 22/40x, area: 2.37 mm^2^, Nikon Eclipse E200 microscope; Nikon, Tokyo, Japan) in the areas with the highest mitotic activity and the average mitotic count/hpf was calculated.

Immunohistochemical phenotyping of lymphomas was conducted according to a routine standard protocol. Pre-treatment for antigen-demasking was performed at a high temperature (96 °C) with EDTA buffer (pH 9.0, HIER T-EDTA pH 9.0, Zytomed #ZUC029-500, Zytomed System GmbH, Berlin, Germany) in a conventional steam heater for 25 min. Primary monoclonal antibodies (CD3 anti-mouse (F7.2.38), Dako 1 #M7254 and CD20 anti-rabbit, Epria 3 #RB-9013-P1), known to be cross-reacting with canine tissue, were each diluted 1:100 in antibody diluent (Zytomed, #ZUC025-100, Zytomed System GmbH, Berlin, Germany). Sections were incubated with the primary antibodies at room temperature for 60 min. For negative controls, the primary antibody was replaced by a buffer. A commercial detection system, the ZytoChem Plus HRP Polymer Kit (#POLHRP-100, Zytomed System GmbH, Berlin, Germany), was applied for 30 min at room temperature. All slides were finally incubated with a chromogen (DAB, diaminobenzidine tetrahydrochloride, #K3468; Dako Denmark A/S, Glostrup, Denmark) for 10 min at room temperature and counterstained with hemalum. Normal canine lymph node tissue served as the positive control.

### 2.2. miRNA Analysis

Total miRNA was isolated from two 10-µm-thick tissue paraffin sections (not mounted on a slide) using the miRNeasy FFPE Kit (QIAGEN, #217504, Hilden, Germany) according to the manufacturer’s instructions (miRNeasy FFPE Handbook, Version January 2020, Protocol Purification of total RNA, deparaffinization with Xylen). Total miRNA was eluted in 40 µL of RNase-free water and diluted 1:5 with RNase-free water. Expression analysis was carried out by ddPCR using specific microRNA assays (Thermo Fisher Scientific, #4427975, Waltham, MA, USA). Reverse transcription was performed using the TaqMan^TM^ MicroRNA Reverse Transcription Kit (Thermo Fisher Scientific, #4366597, Waltham, MA, USA) according to the manufacturer’s instructions (https://www.thermofisher.com/order/catalog/product/4366597, accessed on 3 February 2023) with the specific primer from the TaqMan^TM^ miRNA assay (Thermo Fisher Scientific, #4427975, Waltham, MA, USA). The ddPCR was run on the QX200 Droplet Digital System (Bio-Rad, Hercules, CA, USA) using ddPCR supermix for probes (Bio-Rad, Hercules, CA, USA) and a specific TaqMan^TM^ miRNA assay (Thermo Fisher Scientific, #4427975, Waltham, MA, USA).

All miRNAs described as being mis-regulated in intestinal T-cell lymphoma in dogs (miR-192, 194, 141, 203, 20b, 18b and 363) [16] were screened for the availability of a specific commercially available miRNA assay. TaqMan^TM^ miRNA assays were tested for their functionality in ddPCR. Finally, miR-20b (Thermo Fisher Scientific, assay ID 001014), miR-18b (assay ID 001009), miR-192 (assay ID 000493) and miR-194 (assay ID 000493) were chosen as targets, reflecting two up-regulated (probably oncogenic) and two down-regulated (probably tumour-suppressive) miRNAs. Furthermore, miR-126 (assay ID 000451) and miR-214 (assay ID 002306) were measured, as they have been described as potential general biomarkers for neoplastic diseases [10,24]. Additionally, the RNU6B (assay ID 001973) value was determined and used for normalisation.

An amount of 10 µL cDNA was added to an 11 µL ddPCR supermix and 1 µL TaqMan^TM^ miRNA assay. The generation of droplets was carried out in 8-well cartridges: 20 µL of the abovementioned mix of cDNA, ddPCR supermix and miRNA assay was added to one well, 70 µL oil was added to the destined well. Droplets were generated with the Droplet Maker (Bio-Rad, Hercules, CA, USA) and transferred to a 96-well plate. After sealing the plate, PCR was run with 35 cycles of 94 °C for 30 s, 60 °C for 30 s and 72 °C for 30 s. Measurements were carried out using the QX200 Droplet Reader and QuantaSoft software (Bio-Rad, Hercules, CA, USA). Each PCR was carried out in duplicate; means were used for further process. The normalised values were calculated by building the quotient miR/U6. The validity of our test system was checked by inter- and intra-assay tests as being robust with a variation coefficient of less than 0.15.

### 2.3. Statistics

Statistical analysis of all of the included cases was performed with SAS^®^ 9.4 (TS1M6) statistics for Microsoft Windows. The Kolmogorov-Smirnov test was used to test the data for normal distribution. The *p*-values were adjusted for multiple testing using the Bonferroni correction. Comparison between the groups was carried out using the Mann-Whitney-U test. It was considered statistically significant if *p*-values were <0.05.

## 3. Results

### 3.1. Pathological Characterisation of the Intestinal Findings

#### 3.1.1. Control Cases (Groups 1S and 1L)

**Figure 1:**
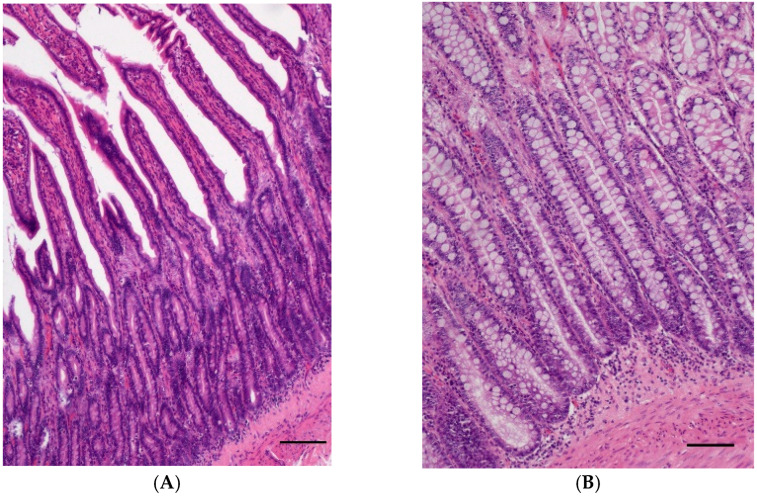
(**A**) Normal intestinal mucosa of the jejunum of a 10-year-old male castrated West Highland White Terrier; (HE, bar = 200 µm). (**B**) Normal intestinal mucosa of the rectum of a 7-year-old female neutered Airedale Terrier; (HE, bar = 200 µm).

Normal small (*n* = 10) or large intestinal tissue samples (*n* = 7) with only few mucosal lymphocytes and plasma cells or mild lymphoplasmacytic infiltration (Figure 1A,B) served as controls. Most controls were full-thickness samples taken from tumour-free or non-inflamed intestinal margins. Only two cases were mucosal biopsies from a submitted organ panel where the intestinal biopsy did not yield any relevant findings.

#### 3.1.2. Enteritis Cases (Groups 2S-a, 2S-b and 2L)

**Figure 2:**
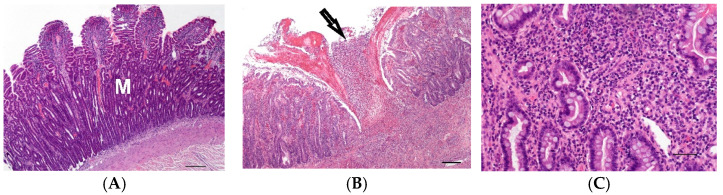
(**A**) Moderate lymphoplasmacytic infiltration and villus atrophy of the mucosa (M) of the jejunum of a 2-year-old male Magyar Vizsla; (HE, bar = 200 µm). (**B**) Focal severe acute ulcerative inflammation of the mucosa (arrow) and submucosa of the ileum in transmural intestinal foreign body disease of a 4-year-old male castrated Belgian Shepherd; (HE, bar = 200 µm). (**C**) Diffuse moderate infiltration of the large intestinal mucosa with lymphocytes, plasma cells and neutrophils in the large intestine of a 4-year-old female Rottweiler; (HE, bar = 50 µm).

To investigate the effect of inflammation, small intestinal samples with moderate to severe lymphoplasmacytic infiltration of the mucosa and moderate villus atrophy suspected to be caused by food intolerance or inflammatory bowel disease were examined (2S-a, *n* = 6, Figure 2A).

Furthermore, seven cases with severe transmural nodular inflammation were analysed (2S-b). In six of these seven cases, foreign body disease was suspected. Acute inflammation in three cases was characterised by ulcerated mucosa with purulent-necrotising inflammation reaching into the muscularis (Figure 2B). In three cases with chronically active transmural inflammation, granulation tissue and pyogranulomatous areas were present. In addition, one terrier had severe lipogranulomas within the tunica muscularis and mesentery due to protein-losing enteropathy.

From the large intestine (2L), five cases were selected which showed a moderate or severe mixed cellular inflammation of the mucosa with erosions, lymphocytes, plasma cells and neutrophils (Figure 2C). The suspected cause was an infectious colitis or inflammatory bowel disease.

#### 3.1.3. Lymphoma Cases (Groups 3S and 3L)

**Figure 3:**
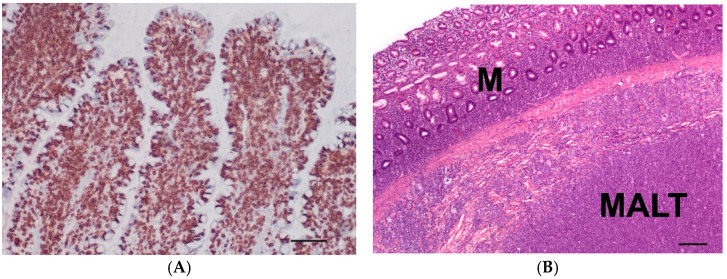
(**A**) Immunohistochemistry of a T-cell lymphoma (small intestine) with intense CD3 expression of neoplastic lymphocytes (brown) of an 11-year-old male castrated mix; (bar = 100 µm). (**B**) Lymphoma in the large intestine affecting the mucosa (M) and mucosa-associated lymphatic-tissue (MALT) of a 5-year-old male Bulldog; (HE, bar = 200 µm).

Small intestinal T-cell lymphoma (3S, *n* = 15) (Figure 3A) had 1–4 mitoses/hpf (mean: 2 mitoses/hpf) and small (*n* = 4) intermediate (*n* = 6) or large (*n* = 5) tumour cells. In all cases, transmural tumour cell infiltration was present. In three cases, the mucosa lost organ-specific structures due to severe infiltration of neoplastic cells. The luminal epithelium showed severe ulcerative inflammation and ulceration in two cases.

Large intestinal B-cell lymphomas (3L, *n* = 12) had 1–6 mitoses/hpf (mean: 3 mitoses/hpf) and had small- (*n* = 2), intermediate- (*n* = 7) or large (*n* = 3)-sized tumour cells. All cases were transmural lymphomas and affected the mucosa-associated lymphatic tissue (MALT) (Figure 3B). The luminal epithelium was either intact or showed erosions.

#### 3.1.4. Carcinoma Cases (Groups 4S and 4L)

**Figure 4:**
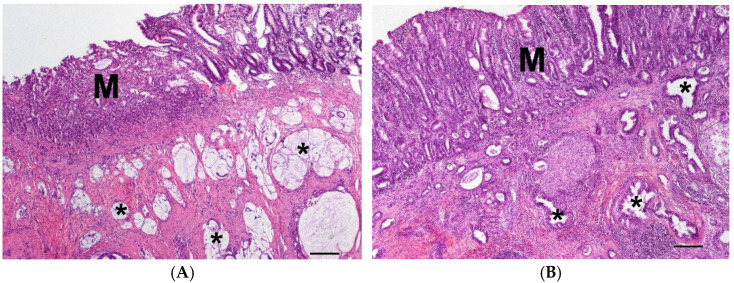
(**A**) Carcinoma in the jejunum with ulcerated mucosa (M) and mucinous neoplastic growth pattern (asterisk) in the muscularis of a 12-year-old male dog of unknown breed; (HE, bar = 200 µm). (**B**) Carcinoma in the large intestine with moderate inflammation of the mucosa (M) and tubular neoplastic growth pattern (asterisk) in the muscularis of a 10-year-old male mix; (HE, bar = 200 µm).

The carcinomas of the small intestine (4S, *n* = 14) had a tubular (*n* = 7), mucinous (*n* = 6, Figure 4A) or solid (*n* = 1) growth pattern and infiltrated the muscularis. The mucosa was ulcerated and moderate to severe inflammation could be seen.

The carcinomas of the large intestine (4L, *n* = 13) mainly had a tubular (*n* = 7, Figure 4B), papillary (*n* = 3), mucinous (*n* = 2) or anaplastic (*n* = 1) growth pattern and infiltrated the muscularis. The mucosa was ulcerated and mild to moderate mixed cellular inflammation could be seen.

### 3.2. miRNA Levels and Correlation between the Groups

**Figure 5:**
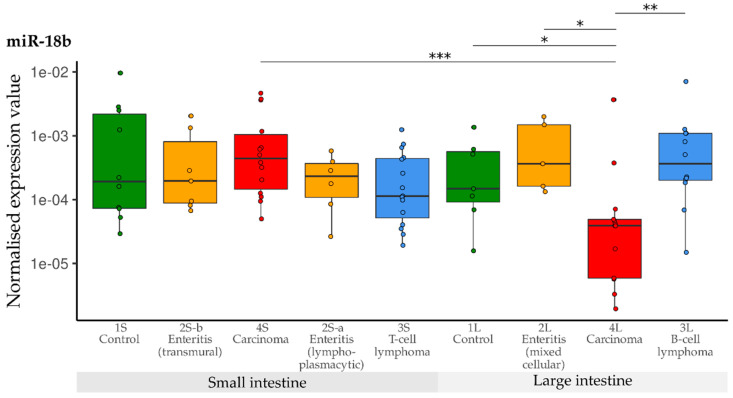
Expression levels of miR-18b in different groups (control, enteritis, carcinomas, lymphomas of the small and large intestine). The absolute miRNA copy number was related to the internal normaliser RNU6B. Results are shown as normalised expression values with a log10 scale. * *p* < 0.05, ** *p* < 0.005, *** *p* < 0.0005. Green control group, yellow enteritis, red carcinoma, blue lymphoma.

In general, expression of miR-18b was significantly lower in the large intestinal carcinoma group than in the small intestinal carcinoma group (*p* = 0.0005, Figure 5). Expression of miR-18b did not differ between any of the small intestine groups. In contrast, significant down-regulation was observed in the large intestinal carcinoma group (4L) compared with the other large intestine groups (1L, 2L, 3L).

**Figure 6:**
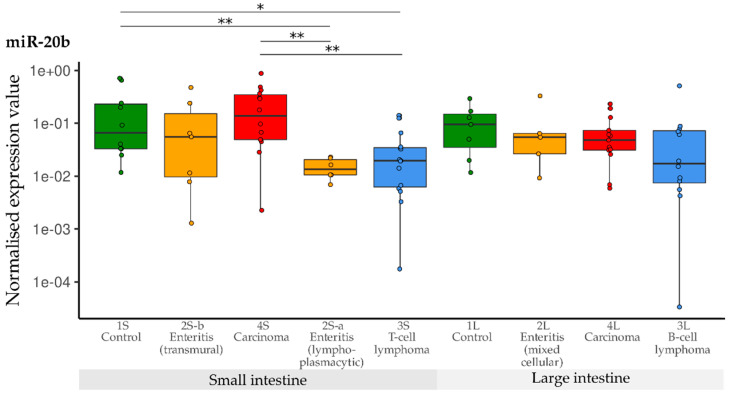
Expression levels of miR-20b in different groups (control, enteritis, carcinomas, lymphomas of the small and large intestine). The absolute miRNA copy number was related to the internal normaliser RNU6B. Results are shown as normalised expression values with a log10 scale. * *p* < 0.05, ** *p* < 0.005. Green control group, yellow enteritis, red carcinoma, blue lymphoma.

Compared with the controls, a significant down-regulation of miR-20b was observed in the group with small intestinal lymphoplasmacytic enteritis (2S-a), as well as in the group with small intestinal T-cell lymphoma (3S). Expression of miR-20b in the carcinoma group (4S) was significantly higher than in the groups with lymphoplasmacytic enteritis (2S-a) and lymphoma (3a)—but did not differ from the controls. Contrary to this, levels of miR-20b did not differ between any of the large intestine groups (1L, 2L, 3L, 4L) (Figure 6).

**Figure 7:**
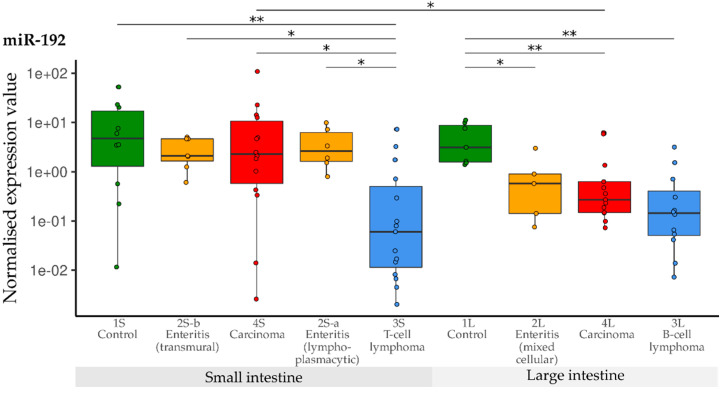
Expression levels of miR-192 in different groups (control, enteritis, carcinomas, lymphomas of the small and large intestine). The absolute miRNA copy number was related to the internal normaliser RNU6B. Results are shown as normalised expression values with a log10 scale. * *p* < 0.05, ** *p* < 0.005. Green control group, yellow enteritis, red carcinoma, blue lymphoma.

Levels of miR-192 were significantly lowered in the T-cell lymphoma group (3S) compared with all other small intestine groups (1S, 2Sa, 2S-b, 4S). Expression of miR-192 was again significantly decreased in all three large intestine groups (2L, 3L, 4L) compared with the controls (1L). The levels were lowest in B-cell lymphomas (3L). Furthermore, expression of miR-192 was higher in small than in large intestinal carcinomas (Figure 7).

**Figure 8:**
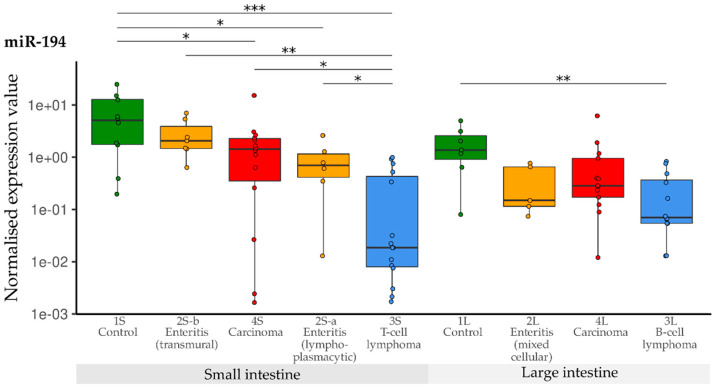
Expression levels of miR-194 in different groups (control, enteritis, carcinomas, lymphomas of the small and large intestine). The absolute miRNA copy number was related to the internal normaliser RNU6B. Results are shown as normalised expression values with a log10 scale. * *p* < 0.05, ** *p* < 0.005, *** *p* < 0.0005. Green control group, yellow enteritis, red carcinoma, blue lymphoma.

When compared with the controls, expression of miR-194 was significantly lower in the small intestine groups 2S-a, 3S and 4S. Only the levels in transmural enteritis cases (2S-b) were in the normal range. In the large intestine groups, down-regulation of miR-194 was observed in B-cell lymphoma (3L) when compared with control tissue samples (1L) (Figure 8).

**Figure 9:**
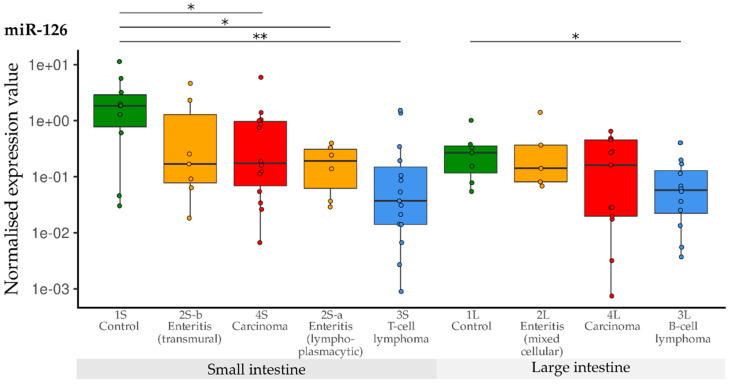
Expression levels of miR-126 in different groups (control, enteritis, carcinomas, lymphomas of the small and large intestine). The absolute miRNA copy number was related to the internal normaliser RNU6B. Results are shown as normalised expression values with a log10 scale. * *p* < 0.05, ** *p* < 0.005. Green control group, yellow enteritis, red carcinoma, blue lymphoma.

Compared with the controls, expression of miR-126 was significantly lower in the groups with lymphoplasmacytic enteritis (2S-a), lymphoma (3S) and carcinoma (4S) of the small intestine. The expression in the group with transmural enteritis (2S-b) was also down-regulated compared with the controls, but significance was not reached due to the different variance. Compared with the controls (1L) in the large intestine groups, significant down-regulation of miR-126 was only observed in B-cell lymphomas (3L) (Figure 9).

**Figure 10:**
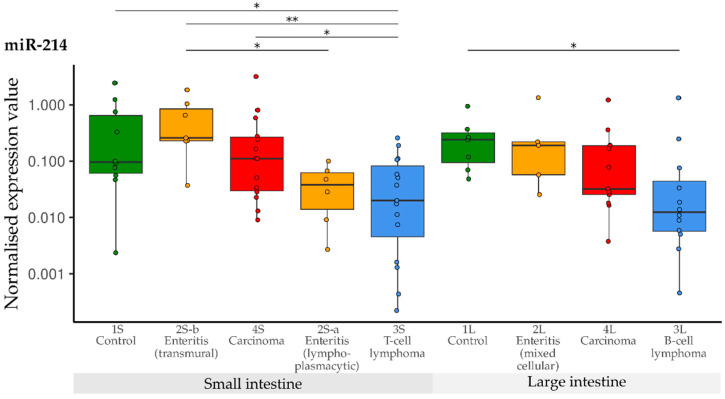
Expression levels of miR-214 in different groups (control, enteritis, carcinomas, lymphomas of the small and large intestine). The absolute miRNA copy number was related to the internal normaliser RNU6B. Results are shown as normalised expression values with a log10 scale. * *p* < 0.05, ** *p* < 0.005. Green control group, yellow enteritis, red carcinoma, blue lymphoma.

Compared with the controls, down-regulation of miR-214 was observed in the T-cell lymphoma group (3S). Furthermore, down-regulation was significant in the T-cell lymphoma group (3S) compared with the transmural enteritis (2S-b) and carcinoma group (4S) of the small intestine. In the lymphoplasmacytic enteritis group (2S-a), levels of miR-214 were lower than in the transmural enteritis group (2S-b). In the large intestine groups, significant down-regulation was only observed in B-cell lymphomas (3L) in comparison with the controls (1L) (Figure 10).

### 3.3. Comparison of miRNA Levels between the Groups (1–4)

#### 3.3.1. Small Intestine vs. Large Intestine (Controls, Carcinoma, Lymphoma)

In the control groups of the small (*n* = 9) and the large (*n* = 7) intestine, there was a trend towards higher expression of miR-126 in small intestinal tissue. Expression levels of miR-18b, 20b, 214, 192 and 194 were not significantly different between the small and large intestine.

In the carcinoma groups (4S, 4L), expression of miR-192 (*p* = 0.0494) and miR-18b (*p* = 0.0005) were significantly lower in large intestinal carcinoma samples (*n* = 13) than in small intestinal carcinoma samples (*n* = 14). The levels of other miRNAs were not different. Growth pattern or secondary inflammation did not affect the miRNA levels.

No significant differences in miRNA levels were observed between T-cell lymphomas of the small intestine (3S, *n* = 15) and B-cell lymphomas of the large intestine (3L, *n* = 12). Cell size or mitotic count, as well as secondary inflammation, did not affect the miRNA levels.

The enteritis groups (2S-a, 2S-b and 2L) were so heterogeneous in cell type that a comparison based on the anatomical site was not made.

#### 3.3.2. Inflammation vs. Controls or Tumour

The lymphoplasmacytic enteritis group (2S-a) was compared with the T-cell lymphoma group (3S), to investigate whether lymphoplasmacytic infiltration could have an impact as a transitional stage: Compared with the normal small intestine (1S), the expression levels of miR-20b, 126 and 194 were significantly lower in cases of lymphoplasmacytic enteritis. Interestingly, this effect was pronounced in cases of lymphomas. Additionally, levels of miR-192 and 214 were significantly lower in T-cell lymphomas compared with the controls and with cases of lymphoplasmacytic enteritis (Table 2).

Carcinomas are often severely ulcerated and inflamed. Thus, the transmural pyogranulomatous enteritis group 2S-b served as a clinically relevant differential diagnosis of nodular masses of the small intestine and to study the effect of ulceration and inflammation as seen in intestinal carcinomas (group 4S). In the small intestine, no differences between the control (1S) and the transmural enteritis group (2S-b) were found. A significant down-regulation of miR-126 and miR-194 could be observed in small intestinal carcinomas (4S) compared with the controls.

In the large intestine, down-regulation was observed for miR-192 in the enteritis group (2L) compared with the controls (1L), and was even more pronounced in the carcinoma group (4L) (Table 3). Expression of miR-18b was significantly lowered in the large intestinal carcinoma group (4L) when compared with the controls (1L), the mixed cellular enteritis group (2L) as well as the group with B-cell lymphoma (3L).

## 4. Discussion

In the present study, the miRNA profiles of canine paraffin-embedded normal, inflammatory and neoplastic tissue of the small and large intestine were characterised. T-cell lymphoma, carcinoma, lymphoplasmacytic and transmural pyogranulomatous enteritis of the small intestine, as well as B-cell lymphoma, carcinoma and mixed enteritis cases of the large intestine, were investigated. We focused on the expression of selected miRNAs: four miRNAs were chosen because they have been described as being mis-regulated in intestinal T-cell lymphoma in dogs with the highest fold change—miR-192 and 194 were down-regulated, while miR-18b and 20b were up-regulated [16]. Two further miRNAs, miR-126 and 214, were measured because they have been described as biomarkers for neoplastic diseases such as osteosarcomas and haemangiosarcomas [10,24].

We confirmed that ddPCR using TaqMan miRNA assays is a robust technique for quantifying miRNA in canine FFPE samples, as previously shown in human and feline samples [30,31,32]. However, the isolation and quantification of miRNAs from FFPE material instead of fresh tissue is debatable. Formalin is known to denature nucleic acids, but FFPE samples are often used in genetic research since they can be easily archived for years, while fresh tissue is rarely available. Using an appropriate miRNA isolation and normalisation protocol seems to overcome these problems and concordance between miRNA data from fresh-frozen and FFPE material can be achieved [33,34,35,36]. Another challenge in miRNA quantification from FFPE can be different fixation protocols, transport times to the laboratory and archiving times. The oldest sample in our study dated back to 2014, 30 samples were collected between 2015 and 2019 and 58 cases were submitted from 2021 to 2022. However, no conspicuous differences in miRNA levels concerning the age of the FFPE material were observed (data not shown).

Another critical point in the quantification of miRNA from FFPE material is the normalisation strategy. As quantification of miRNA by ddPCR is a relatively new approach, no generally accepted procedure for normalisation has been published. Normalisation is needed as the amount of tissue, and thus the number of cells, varies between samples—depending on the size of the embedded material. Therefore, normalisation is carried out by relating the miRNA levels to a “housekeeping gene”, such as RNU6B, which is assumed to be equally expressed in all cells. As an alternative, another uniformly expressed miRNA could be determined with a software such as NormFinder and used for normalisation. In FFPE samples, using RNU6B for normalisation is the most common method in quantitative real-time PCR and ddPCR [16,37], but it is under discussion [38,39].

First, we compared levels of miRNAs between the small and large intestine: In control tissue, expression of miR-126 was slightly higher in the small intestine than in the large intestine, while the other miRNAs were equally expressed. In carcinomas, we observed a significantly lower expression of miR-192 and highly significant down-regulation of miR-18b expression in the large intestine compared with the small intestine. Because of these differences, we decided to analyse the groups according to anatomical site instead of summarising all carcinomas, lymphomas and control samples. These findings underline the importance of carefully selecting suitable controls and case groups, taking into account various factors.

Second, we investigated the miRNA profile of normal tissue compared with the different inflammatory and neoplastic diseases. We expected the two probably oncogenic miRNAs (miR-18b and 20b) to be up-regulated, as it has been described in canine T-cell lymphomas [16] and single samples of feline carcinomas, as well as T- and B-cell lymphomas [32]. Surprisingly, we did not find any mis-regulation of miR-18b in the T-cell lymphomas of the small intestine, or even down-regulation of miR-20b in the T-cell lymphomas and cases of lymphoplasmacytic enteritis of the small intestine. Additionally, we detected lower expression of miR-18b in the large intestinal carcinoma group, which is in contrast the described up-regulation of miR-18b in human colorectal cancer cell lines [17]. According to the literature, miR-18b and 20b are known to be part of the pro-oncogenic miR-106a-363 polycistronic cluster [40,41]. However, their role is not yet fully understood as there are also reports of tumour-suppressive functions: expression of miR-20b was downregulated in different human colorectal tumours [42], and transfection of human oral squamous carcinoma cells with miR-20b resulted in decreased proliferation [43]. Additionally, even though miR-20b is part of the miR-106a-363 cluster, it is not over-expressed in human T-cell leukaemia, in contrast to the other members of the cluster [40]. These conflicting findings suggest that miRNAs may have tissue-specific and sometimes contradictory functions [44]. Not to forget, discrepancies between different studies may be explained by different quantification methods (qPCR vs. ddPCR) and/or normalisation strategies [38]. The selection of samples is also a critical point when comparing studies, so distinguishing between lymphoma subtypes can cause differences in miRNA expression values [16].

Both putative tumour-suppressive miRNAs (miR-192 and miR-194) were down-regulated in T-cell lymphomas and lymphoplasmacytic enteritis of the small intestine, as well as in B-cell lymphomas of the large intestine. This corresponds to the finding that miR-192 and 194 are down-regulated in intestinal T cell lymphomas [16]. Additionally, miR-192 was down-regulated in the carcinoma group of the large intestine. This supports the hypothesis of miR-192 playing an important role in the sequential development of human colorectal adenomas [20]. In dogs, too, miR-192 seems to play a role in the progression of diseases related to the large intestine [19]. In contrast, in cats, miR-192 was found up-regulated in carcinoma and some B-cell lymphoma samples of the small intestine [32]. Since there have also been studies in humans describing miR-192 as being up-regulated [45,46] or down-regulated [46], the role of miR-192 in different types of tumours appears contentious. Similar to miR-192, miR-194 was down-regulated in small intestinal carcinomas, supporting the findings of tumourigenicity of intestinal tumour organoids [21]. Furthermore, miR-194 serum levels were lowered in human intestinal-type gastric cancer [22] and FFPE levels of miR-194 were down-regulated in human colorectal adenomas [23]. Overall, miR-194 seems to play a crucial role in different intestinal neoplasms.

In several canine endothelial and epithelial neoplastic diseases, miR-126 and miR-214 were described as being upregulated when analysed as serological/circulating markers [10,24]. Therefore, a certain relevance in all types of cancer was postulated. However, levels of circulating miR-214 were not significantly higher in adenocarcinomas and lymphomas; only levels of miR-126 in adenocarcinomas were significantly increased [10]. In contrast, in our study, miR-126 was expressed to a lower extent in the groups with carcinoma, lymphoma and lymphoplasmacytic enteritis of the small intestine, implying tumour-suppressive function rather than oncogenicity. Additionally, in humans, miR-126 has been described as having a tumour-suppressive function: It was down-regulated in colorectal cancer [47] and colorectal cancer cells [48]. Artificial over-expression of miR-126 in colon cancer cells led to the inhibition of cell viability, cell migration and invasion capacity [49]. Even in mice, it was shown that miR-126 exhibits an anti-tumour effect by activating macrophages and altering the proliferation and the migration of tumour cells [50]. These findings indicate that down-regulation of miR-126 in tissue is correlated with cancer development. Additionally, in our study, levels of miR-126 were lowered in B-cell lymphomas of the large intestine and were not altered in the carcinoma and enteritis samples of the large intestine. In both T-cell lymphomas of the small intestine and B-cell lymphomas of the large intestine, miR-214 was down-regulated. All three types of neoplasms are characterised by the fact that the majority of the cells present are lymphocytes. This could explain why there is a similar change in miRNA expression. Discrepancies between our findings and the studies by Heishima et al. [10,24] can be ascribed to the different miRNA sources—serum and tissue. A difference between tumour profile and circulating miRNAs has already been described for splenic tumours [51] and lymphoma samples [52]. Not all miRNAs might be released into the serum, some may only be released but not stored in cancer cells, serum miRNA levels might be too low to change the total amount of circulating miRNA and neighbouring cells could release miRNA because of neoplasms [51].

Third, we focused on the clinical relevance of the miRNA shifts, since it is crucial for prognosis and treatment to be able to differentiate between transmural enteritis, carcinoma and lymphoma. In the small intestine, transmural enteritis clearly differed from carcinoma cases, since no miRNA was mis-regulated in cases of enteritis compared with the controls, while miR-126 and miR-194 were down-regulated in the carcinoma cases. Additionally, clear discrimination was possible between the two enteritis groups since miR-20b, miR-126 and miR-194 were down-regulated in cases of lymphoplasmacytic enteritis. This down-regulation was even more pronounced in the T-cell lymphoma group, with miR-214 and miR-192 also showing reduced expression. Both neoplasms are characterised by the fact that the majority of the cells present are lymphocytes. This could explain the similar change in miRNA expression. Furthermore, these changes possibly imply that lymphoplasmacytic infiltration could be a relevant transitional stage towards lymphoma. Distinguishing between enteritis and lymphoma by means of miR-192 and miR-214 would be possible. Overall, a differentiation of the case groups of the small intestine can be achieved by using the miRNA profile of the investigated miRNAs.

In the cases of mixed cellular enteritis of the large intestine, only miR-192 was down-regulated. This change was even more pronounced in the carcinoma group, with miR-18b also being lowered. These findings might indicate that enteritis plays a role as a transitional state from normal to cancer tissue. B-cell lymphoma of the large intestine showed down-regulation of miR-126, miR-214, miR-192 and miR-194, and differed from carcinoma of the large intestine (miR-18b lower in carcinoma), as well as from T-cell lymphoma of the small intestine (miR-20b not mis-regulated).

Although it seems possible to distinguish cases based on the miRNA profile of tissue using ddPCR, further investigations are necessary: The small number of cases and controls in each group has to be increased to strengthen the statistical power and to define relevant thresholds for diagnostic approaches. The aim should be to find suitable biomarkers for use in liquid biopsy so that invasive surgery can be avoided. For this purpose, it is important to investigate how expression changes of these miRNAs in serum samples can be detected. Since it is uncertain whether there is a correlation between tissue and serum levels, focusing on altered miRNAs in tissue when analysing serum does not seem to be the right way. Instead, screening tests (miRNA sequencing or arrays) should be used to search for biomarkers in serum, so as not to miss relevant miRNA changes.

## 5. Conclusions

In summary, we showed that ddPCR is a suitable technique for quantifying miRNA in canine FFPE material. Differences in miRNA profiles between large and small intestinal tissues were described. Furthermore, distinct miRNA patterns were observed, making it possible to discriminate between normal, enteritis, carcinoma and lymphoma cases of the small and large intestine. In the small intestine, miR-126 and 194 were down-regulated in carcinomas, while miR-20b, 126, 214, 192 and 194 were down-regulated in T-cell lymphomas. In the large intestine, miR-192 and 18b were down-regulated in carcinomas, while miR-126, 214, 192 and 194 were down-regulated in B-cell lymphomas. Further investigations are needed for using circulating miRNAs as biomarkers, since different neoplasms seem to lead to different changes that are not always reflected in both tissue and serum.

## Figures and Tables

**Table 1:** Signalment of the dogs included in the miRNA analysis study (*n* = 89).

Group	Breed	Age (Years)	Sex
Group 1S: controls small intestine (n = 10)	4 Terrier, 2 mix, 1 Dachshund, 1 French Bulldog, 1 Pug, 1 Rhodesian Ridgeback	2–15	3 m, 4 mc, 3 fn
Group 1L: controls large intestine (n = 7)	2 Labrador, 1 Akita, 1 Hovawart, 1 mix, 1 Munsterlander, 1 Terrier	4–10	3 m, 1 f, 3 fn
Group 2S-a: enteritis (lymphoplasmacytic) of the small intestine (n = 6)	2 mix, 1 Beagle, 1 French Bulldog, 1 Magyar Vizsla, 1 Rottweiler	2–10	3 m, 1 mc, 2 fn
Group 2S-b: enteritis (transmural) of the small intestine (n = 7)	2 Shepherd, 1 Labrador, 1 mix, 1 Rhodesian Ridgeback, 1 Shar Pei, 1 Terrier	1–11	3 m, 2 mc, 2 f
Group 2L: enteritis (mixed) of the large intestine (n = 5)	2 Shepherd, 1 Labrador, 1 mix, 1 Rottweiler	1–5	2 f, 3 fn
Group 3S T-cell lymphoma of the small intestine (n = 15)	3 mix, 2 Labrador, 2 Terrier, 1 Cocker Spaniel, 1 French Bulldog, 1 Havanese, 1 Magyar Vizsla, 1 pug, 1 Schnauzer, 1 Shepherd, 1 Shih Tzu	7–12	7 m, 3 mc, 3 f, 2 fc
Group 3L: B-cell lymphoma of the large intestine (n = 12)	3 mix, 2 Terrier,1 Bulldog, 1 Dachshund, 1 Husky, 1 Lhasa Apso, 1 Munsterlander, 1 Pointer, 1 Wolfhound	1–12	6 m, 5 f, 1 fn
Group 4S: carcinoma of the small intestine (n = 14)	4 mix, 4 Terrier, 1 Dachshund, 1 Gordon Setter, 1 Kooikerhondje, 1 Pointer, 1 Poodle, 1 pug	7–15	5 m, 4 mc, 1 f, 3 fn, 11 unknown
Group 4L: carcinoma of the large intestine (n = 13)	5 mix, 2 Pointer, 1 Border Collie, 1 Labrador, 1 Rhodesian Ridgeback, 1 Samojede, 1 Shepherd, 1 Terrier	7–13	4 m, 6 mc, 3 fn

Legend: f = female; fn = female neutered; m = male; mc = male castrated; mix = mixed breed; S = small intestine, L = large intestine.

**Table 2:** Significance of expression differences between groups in the small intestineshown as *p* values. Up-regulation is marked with an arrow up, down-regulation is marked with an arrow down.

	1S vs. 2S-b	1S vs. 4S	1S vs. 2S-a	1S vs. 3S	2S-a vs. 2S-b	2S-a vs. 4S	2S-a vs. 3S	2S-b vs. 4S	2S-b vs. 3S	3S vs. 4S
miR-18b	1.000	0.5387	0.7863	0.3317	0.8303	0.1735	0.9070	0.3139	0.4381	0.0636
miR-20b	0.3539	0.4292	**0.0040 ↓**	**0.0158 ↓**	0.3531	**0.0034 ↑**	0.6685	0.2183	0.3976	**0.0018 ↑**
miR-126	0.2617	**0.0498 ↓**	**0.0262 ↓**	**0.0025 ↓**	0.8303	0.5919	0.2276	0.9702	0.0907	0.0576
miR-214	0.0927	0.6605	0.0927	**0.0247 ↓**	**0.0124 ↑**	0.1078	0.8457	0.1087	**0.0024 ↓**	**0.0246 ↑**
miR-192	0.4642	0.5387	0.6255	**0.0043 ↓**	0.8303	0.9671	**0.0072 ↓**	0.9702	**0.0074 ↓**	**0.0083 ↑**
miR-194	0.4068	**0.0434 ↓**	**0.0448 ↓**	**0.0003 ↓**	0.0538	0.4333	**0.0391 ↓**	0.2183	**0.0006 ↓**	**0.0064 ↑**

Legend: 1S = control small intestine; 2S-a = lymphoplasmacytic enteritis small intestine; 2S-b = transmural enteritis small intestine; 3S = T-cell lymphoma small intestine; 4S = carcinoma small intestine; significant down-regulation (arrow down); significant up-regulation (arrow up); light red *p* < 0.05; red *p* < 0.005; dark red *p* < 0.0005.

**Table 3:** Significance of expression differences between groups in the large intestine shown as *p* values. Up-regulation is marked with an arrow up, down-regulation is marked with an arrow down.

	1L vs. 2L	1L vs. 4L	1L vs. 3L	2L vs. 3L	2L vs. 4L	3L vs. 4L
miR-18b	0.2556	**0.0394 ↓**	0.4220	0.8744	**0.0180 ↓**	**0.0043 ↓**
miR-20b	0.7453	0.4757	0.0832	0.4932	0.9215	0.3143
miR-126	1.000	0.4281	**0.0384 ↓**	0.0651	0.4902	0.4303
miR-214	0.6261	0.0813	**0.0160 ↓**	0.0512	0.2782	0.0866
miR-192	**0.0230 ↓**	**0.0034 ↓**	**0.0027 ↓**	0.2684	1.000	0.1211
miR-194	0.0740	0.0813	**0.0035 ↓**	0.2684	0.4304	0.0684

Legend: 1L = control large intestine; 2L = mixed cellular enteritis large intestine; 3L = B-cell lymphoma large intestine; 4L = carcinoma large intestine; significant down-regulation (arrow down); significant up-regulation (arrow up); light red *p* < 0.05; red *p* < 0.005; dark red *p* < 0.0005.

## Data Availability

The raw data of the results presented in this study are available on request from the corresponding author.

## Abbreviations

ddPCR = droplet digital polymerase chain reaction; f = female; FFPE = formalin-fixed paraffin-embedded; fn = female neutered; m = male; mc = male castrated; mix = mixed breed; S = small intestine, L = large intestine.
